# Concordance of rapid diagnostic test results between health facility registers and health management information systems: a multi-country evaluation

**DOI:** 10.1186/s12936-025-05753-4

**Published:** 2025-12-31

**Authors:** Abibatou Konaté-Touré, Corine Ngufor, Arthur Mpimbaza, Sunday Atobatele, Ese Akpiroroh, Nelson Ssewante, Idelphonse B. Ahogni, Orphée M. A. Kangah Kouakou, Valérie A. Bedia-Tanoh, Jacques Agnon, Cyriaque Affoukou, Bosco Agaba, Onyebuchi Okoro, Michael Humes, Kevin Griffith, Anatole N. N. Mian, Antoine M. Tanoh, Kim A. Lindblade, William Yavo

**Affiliations:** 1 Centre de Recherche et de Lutte contre le Paludisme, Institut National de Santé Publique, BPV 47, Abidjan, Côte d’Ivoire; 2 Centre de Recherche Entomologique de Cotonou, Cotonou, Benin; 3 Child Health and Development Centre, College of Health Sciences, Makerere University, Kampala, Uganda; 4 Sydani Group, Abuja, Nigeria; 5 Programme National de Lutte Contre le Paludisme, Abidjan, Côte d’Ivoire; 6 Programme National de Lutte Contre le Paludisme, Cotonou, Benin; 7 National Malaria Control Division, Kampala, Uganda; 8 National Malaria Elimination Programme, Abuja, Nigeria; 9 U.S. President’s Malaria Initiative, USAID, Washington, DC USA; 10 PMI Insights Project, PATH, Geneva, Switzerland

**Keywords:** Discrepancy, DHIS2, Malaria, Data quality, Surveillance, RDTs

## Abstract

**Background:**

Accurate routine surveillance data are essential for malaria control and elimination. However, the multistep reporting process used in most malaria-affected countries can introduce discrepancies between health facility registers and national health management information systems, often based on the District Health Information System 2 (DHIS2). This study assessed the concordance of malaria rapid diagnostic test (RDT) data across facility registers, monthly summary forms (MSFs) and the DHIS2 in four sub-Saharan countries.

**Methods:**

In 2023, we conducted an observational study in Benin, Côte d’Ivoire, Nigeria, and Uganda, using harmonized tools and methods. In each country, 16 public primary health care facilities were selected from two regions. The total number of RDTs and positive results from health facility registers, MSFs and the DHIS2 were compared over three to five months. We assessed concordance using Bland–Altman plots, weighted absolute percentage error (WAPE)-based aggregate data reporting accuracy (WADRA), and verification factors (VFs). System- and facility-level differences were examined by stratifying indicators by region, baseline outpatient volume and test positivity rate.

**Results:**

Across 64 facilities, 104,396 RDTs (58,304 positives, 55.8%) were recorded in registers, compared to 112,435 (62,903 positives, 55.9%) in MSFs and 110,771 (62,761 positives, 56.7%) in DHIS2. Benin showed the highest concordance across data sources, while Nigeria and Uganda had the lowest. Positive RDT results were more likely to be reported than total RDTs, particularly in Nigeria, where VFs indicated consistent overreporting (mean VF 0.74, 95% CI: 0.61–0.89). WADRA analysis showed low reporting accuracy for positive RDTs in 6 (38%) Nigerian and 5 (31%) Ugandan facilities. Regional differences were notable in Nigeria and Uganda. In Nigeria, higher outpatient volume was associated with lower concordance; no trend was seen for baseline test positivity rate.

**Conclusions:**

Substantial variation in RDT data concordance was observed across countries and facilities. Concordance was strongest between MSFs and DHIS2, suggesting data entry was not a significant issue. Strengthening routine data validation and using accuracy and direction-sensitive metrics, such as WADRA and VFs, could improve malaria data reliability. Further research should explore system-level factors influencing data quality and identify scalable solutions.

**Supplementary Information:**

The online version contains supplementary material available at 10.1186/s12936-025-05753-4.

## Background

Malaria remains a major public health challenge globally, with the greatest burden concentrated in sub-Saharan Africa. Despite ongoing efforts to eliminate transmission, malaria continues to affect 83 countries. In 2023, an estimated 263 million cases and 597,000 deaths occurred worldwide, underscoring the continued impact of the disease on global health [1].

In response to this ongoing burden, the World Health Organization (WHO) has prioritized universal access to malaria prevention, diagnosis, and treatment as the first pillar of the Global Technical Strategy for Malaria (GTS) 2016–2030 [2]. Laboratory confirmation of malaria infection, either by rapid diagnostic tests (RDT) or microscopy, is recommended prior to treatment and is a critical component of effective case management [3]. Beyond its clinical importance, increased use of diagnostic testing has also strengthened the quality of data generated through routine surveillance systems.

Surveillance, defined as the collection, analysis and use of malaria data to plan, implement and evaluate programs, is the third pillar of the GTS and a cornerstone of national malaria strategies [2]. In many malaria-affected countries, routine surveillance data from public outpatient clinics are recorded through a multi-step process: First, patient information, including patient demographics, clinical symptoms, diagnostic test results, diagnoses and treatments prescribed is handwritten into health facility registers, of which there may be several in a facility that represent different departments. Second, data from these registers are manually compiled and aggregated into a monthly summary form (MSF), often completed in duplicate and submitted to the district or equivalent administrative level. Third, in some countries, a data validation step is performed at the district level to review and verify the MSF before it is submitted for entry. Fourth, the validated and aggregated data are manually entered into the national health management information system (HMIS), which is frequently based on the District Health Information Software 2 (DHIS2, Health Information Systems Program, University of Oslo, Norway), an open source, web-based platform used by over 75 low- and middle-income countries to manage and analyze health data [4]. Finally, additional data quality checks may be conducted at the national level to ensure consistency and accuracy before the data are used for analysis and reporting.

This multistep process, from patient-level recording to system-level reporting, introduces multiple opportunities for errors to arise between the original source data and the final data reported on DHIS2. Ensuring the accuracy of these data is critical. High-quality surveillance data are essential for identifying populations most affected by malaria, targeting interventions effectively, and monitoring progress toward control and elimination goals.

Routine data quality assessments play a vital role in ensuring that malaria surveillance systems generate information that is both reliable and actionable. However, such assessments have frequently revealed discrepancies between data recorded in the DHIS2 and original source documents at health facilities [5–8]. Various approaches have been used to assess the concordance between data sources, providing important insights to guide investments in strengthening surveillance systems. This study aimed to evaluate whether data on malaria RDTs recorded in health facility registers, the primary source documents, was correctly summarized in the MSF and accurately reported into the DHIS2 across four sub-Saharan countries, and to explore the effect of facility-level factors on concordance.

## Methods

### Study design

In 2023, we conducted an observational study to assess the accuracy of malaria RDT results recorded in health facility registers and reported to the national HMIS in Benin, Côte d’Ivoire, Nigeria, and Uganda [9]. The objective of the analysis presented in this report was to measure the concordance of data across three steps of the data registration process for two key indicators: the number of RDTs performed and the number of RDT-positive results. The data compared included: health facility registers (outpatient departments and, where applicable, antenatal care registers); the MSF used to aggregate data for reporting to the district (or equivalent); and the HMIS platform, which was based on the DHIS2 in all countries.

To ensure comparability across settings with differences in malaria surveillance systems (Table 1), we harmonized study methods and data collection instruments across all four countries. Data collection for this analysis began in July 2023 in Benin, Nigeria, and Uganda and September 2023 in Côte d’Ivoire, continuing through November 2023 in all countries.

**Table 1:** Characteristics of the malaria surveillance systems by country

	Benin	Côte d’Ivoire	Nigeria	Uganda
Characteristics of health facility registers	Multiple patient records per page	Single patient record per page	Multiple patient records per page	Multiple patient records per page
Types of registers where malaria RDT data are recorded	Antenatal care OPD	Antenatal care OPD	OPD* Laboratory register	OPD Laboratory register
Data management for Health management information system	DHIS2	DHIS2	DHIS2	DHIS2
HMIS data entry point	Health district	Health district	Local government area	Health district

OPD*: Outpatient Department

### Study sites and sampling

In each country, the study was conducted in two geographically distinct areas to reflect variation in malaria seasonality and transmission intensity. Within each area, two lower level administrative areas (admin 2 areas) were purposively selected: health zones (Benin); health districts (Côte d’Ivoire); local governmental areas (Nigeria); and districts (Uganda). Eligible public health facilities were identified using DHIS2 data, including only those with high data completeness (> 75% of monthly reports) and RDT testing volume (> 50 RDTs per month) over the previous 24 months [9]. For each facility, we calculated total outpatient volume and overall RDT positivity over the preceding two years. Facilities were then stratified by median values into four groups based on outpatient volume (high/low) and RDT positivity rate (high/low). One facility was randomly selected from each stratum, resulting in 16 facilities per country (64 total).

### Data collection procedures

Trained research assistants collected data in health facilities using structured electronic questionnaires developed in KoboToolbox (Cambridge, MA, USA) that employed logic and range checks. The tools were translated into French using DeepL (Cologne, Germany) and validated by principal investigators in Benin and Côte d’Ivoire. Study supervisors validated data collection during supervision visits. Details of training and deployment procedures are reported elsewhere [9].

Facility-level characteristics were collected through surveys at the study outset. On a daily basis, research assistants reviewed outpatient and antenatal care registers, summarizing the number and results of RDTs, malaria diagnoses, and antimalarial treatments. At the end of each month, research assistants recorded RDT-related indicators from the MSFs submitted to the district for entry into DHIS2. After the study period, monthly RDT data for each facility were extracted from the country’s DHIS2 instance.

### Data management and analysis

Data management and analysis were conducted using R (R Foundation for Statistical Computing, Vienna, Austria). Numbers and proportions of key descriptive characteristics of the selected health facilities in each country were calculated. Average estimates of *Plasmodium falciparum* parasite prevalence among children aged 2–10 years (PfPR_2–10_) for a 5-km radius around each health facility were extracted from the Malaria Atlas Project using the malariaAtlas package [9].

Reporting completeness was defined as the number of facility-months in which non-zero RDT data were reported to DHIS2, divided by the number of months expected given the study duration in each country.

Two indicators were assessed across the three data sources: the total number of rapid diagnostic tests (RDTs) performed and the number of positive RDT results. These indicators were compared at the level of facility-month observations using three pairwise comparisons: (1) health facility register versus MSF, (2) MSF versus DHIS2, and (3) health facility register versus DHIS2. In comparisons involving the health facility register, the MSF dataset was considered the aggregated data source. In comparisons with DHIS2, the MSF dataset was treated as the source document. No records were excluded due to missing data in the MSF or DHIS2 sources.

Three complementary but different approaches were used to assess concordance between data sources. First, Bland–Altman plots based on percentage differences were generated separately by country, indicator, and comparison to evaluate both bias and precision [10]. Percentage differences were used instead of absolute differences as the latter can be misleading when the number of RDTs performed per month and the positivity rates vary widely. The percentage difference was defined as the difference between the source document and aggregated report divided by their mean and multiplied by 100. Limits of agreement (LoA) were calculated as the mean percentage difference ± 1.96 times the standard deviation of the percentage differences. Wide LoA indicate poor agreement and high variability between data sources.

Second, the weighted absolute percentage error (WAPE) was used to quantify the discrepancy between values recorded in the source documents and those reported in the system data. WAPE is calculated as the sum of the absolute differences between the two sources divided by the total from the source document, and expressed as a percentage. The formula is given as [7]:

$$WAPE = \left(\frac{\sum_{m=1}^{n}\left|{y}_{m-}{\widehat{y}}_{m}\right|}{\sum_{m=1}^{n}{y}_{m}}\right)*100$$

where *n* is the number of months of observation, ${y}_{m}$ is the value of the indicator in the source document for month *m* and ${\widehat{y}}_{m}$ is the corresponding value reported in the aggregated data. WAPE gives greater weights to errors associated with higher volumes of data by scaling each monthly error by the total number of records in the source. Additionally, WAPE penalizes both over- and under-reporting.

To facilitate interpretation, a WAPE-based metric of reporting accuracy, termed the weighted aggregate data reporting accuracy (WADRA), was calculated as:

$$WADRA=100-WAPE$$

Reporting accuracy was classified based on previously published thresholds as low (< 70%), moderate (70–84%), or high (≥ 85%) [7].

Lastly, verification factors (VF) are recommended by the WHO to measure disparities between data sources and are calculated as the ratio of the indicator recorded in the source document to that reported from the aggregated report [11]. VFs indicate the overall direction of differences between data sources, with values > 1 indicating under-reporting and values < 1 indicating over-reporting by the aggregate report. We calculated VFs to compare indicators between the health facility register and the DHIS2 only as:

$${VF}_{m}= \frac{{y}_{m}}{{\widehat{y}}_{m}}$$

where ${y}_{m}$ is the value of the indicator in the register and ${\widehat{y}}_{m}$ is the value of the indicator in the DHIS2 over *m* month. We averaged monthly VFs to calculate the mean VF for the evaluation period for each health facility and indicator. VFs were log-transformed to calculate standard errors and 95% confidence intervals (CI) after which they were back-transformed to the original scale. VFs with point estimates less than 10% over- or underreporting (i.e. 0.9 ≤ VF < 1.1) and 95% confidence intervals including 1 were considered to indicate no serious reporting concerns [11].

In countries where concordance between data sources varied across health facilities, we examined whether region, baseline outpatient volume, TPR or PfPR_2–10_ were associated with differences in concordance. To do this, we stratified WADRA and VFs according to region, baseline strata for outpatient volume and TPR and PfPR_2–10_ for both indicators.

### Ethical issues

The PATH institutional review board approved the multi-country study. In Benin, the Comité National d’Ethique pour la Recherche en Santé provided approval. In Côte d’Ivoire, the Comité National d’Éthique des Sciences de la Vie et de la Santé reviewed and approved the study. In Nigeria, approval was received from Oyo State Ministry of Health Research Ethics Committee, Sokoto State Health Research Ethics Committee and the National Health Research Ethics Committee of Nigeria. The Uganda National Council for Science and Technology and Vector Control Division-Research & Ethics Committee both reviewed and approved the Uganda study.

## Results

In Benin, Côte d’Ivoire and Nigeria, all selected facilities were basic public, primary healthcare facilities providing only outpatient and antenatal services (Table 1). In Uganda, selected health facilities were a mix of level II (i.e. basic primary health care) and level III (health units with laboratory and maternal healthcare services). Most facilities in Benin were located in higher transmission areas (PfPR_2–10_ ≥ 30%) whereas the majority of facilities in the other three countries were located in areas where PfPR_2–10_ was less than 30%. Across the four countries, most facilities had at least three staff performing RDTs. Whereas Côte d’Ivoire reported no laboratory technicians at any of its facilities, the other countries reported between 4 and 11 facilities out of 16 with laboratory technicians (Table 2).

**Table 2:** Characteristics of participating health facilities by country, 2023

	Benin N = 16 n (%)	Côte d’Ivoire N = 16 n (%)	Nigeria N = 16 n (%)	Uganda N = 16 n (%)
Type of establishment				
Level 1	16 (100)	16 (100)	16 (100)	0 (0)
Level 2	0 (0)	0 (0)	0 (0)	10 (62.5)
Level 3	0 (0)	0 (0)	0 (0)	6 (37.5)
*P. falciparum* parasite rate (PfPR_2–10_)				
0–9%	0 (0)	0 (0)	0 (0)	1 (6.2)
10–19%	0 (0)	2 (12.5)	7 (43.8)	1 (6.2)
20–29%	1 (6.2)	9 (56.3)	7 (43.8)	9 (56.3)
30–39%	15 (93.8)	5 (31.3)	2 (12.5)	5 (31.3)
Number of staff performing RDTs				
1–2	1 (6.2)	0 (0)	3 (18.8)	2 (12.5)
3–4	4 (25.0)	4 (25.0)	6 (37.5)	4 (25.0)
5 or more	11 (68.8)	12 (75.0)	7 (43.8)	10 (62.5)
Laboratory technician present	4 (25.0)	0 (0)	11 (68.8)	7 (43.8)

Data reporting completeness in the DHIS2 was high for selected facilities across all four countries. Côte d’Ivoire achieved 100% reporting completeness with 48 monthly reports available over the three month study period for across 16 health facilities. Benin and Nigeria reported 98.75% completeness (79 reports out of 80 expected), while Uganda reported 97.5% (78 reports out of 80 expected).

Across all study sites, a total of 104,396 RDTs were recorded from facility registers, of which 58,304 (55.8%) were reported as positive. From the MSFs, the study team documented 112,435 RDTs, with 62,903 (55.9%) reported as positive. DHIS2 data showed 110,771 RDTs performed and 62,761 positive results, yielding a test positivity rate of 56.7%. Monthly totals of RDTs performed and positive results, disaggregated by country and data source, are presented in Table S1 (Additional File 1).

### Bland–Altman plots

**Fig. 1:**
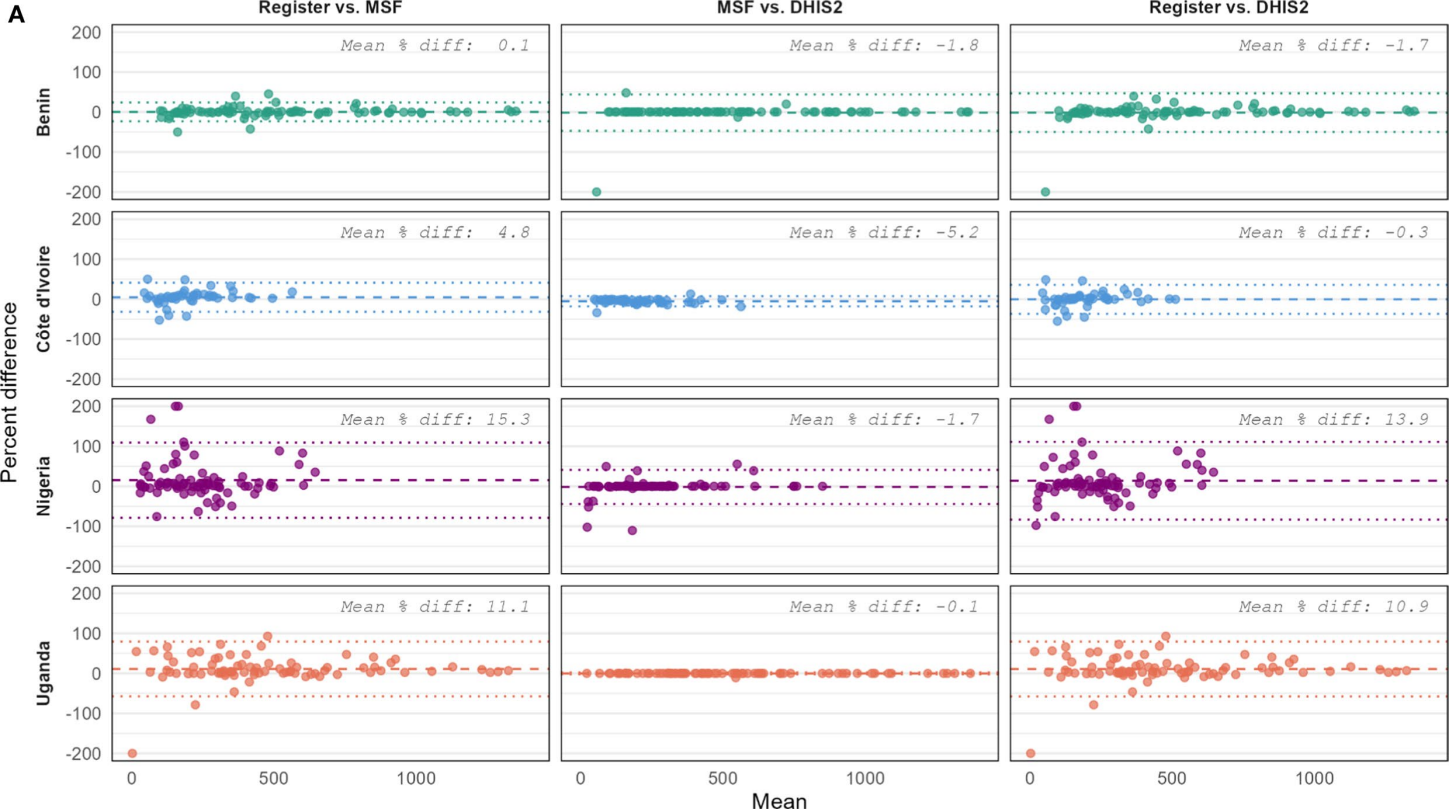

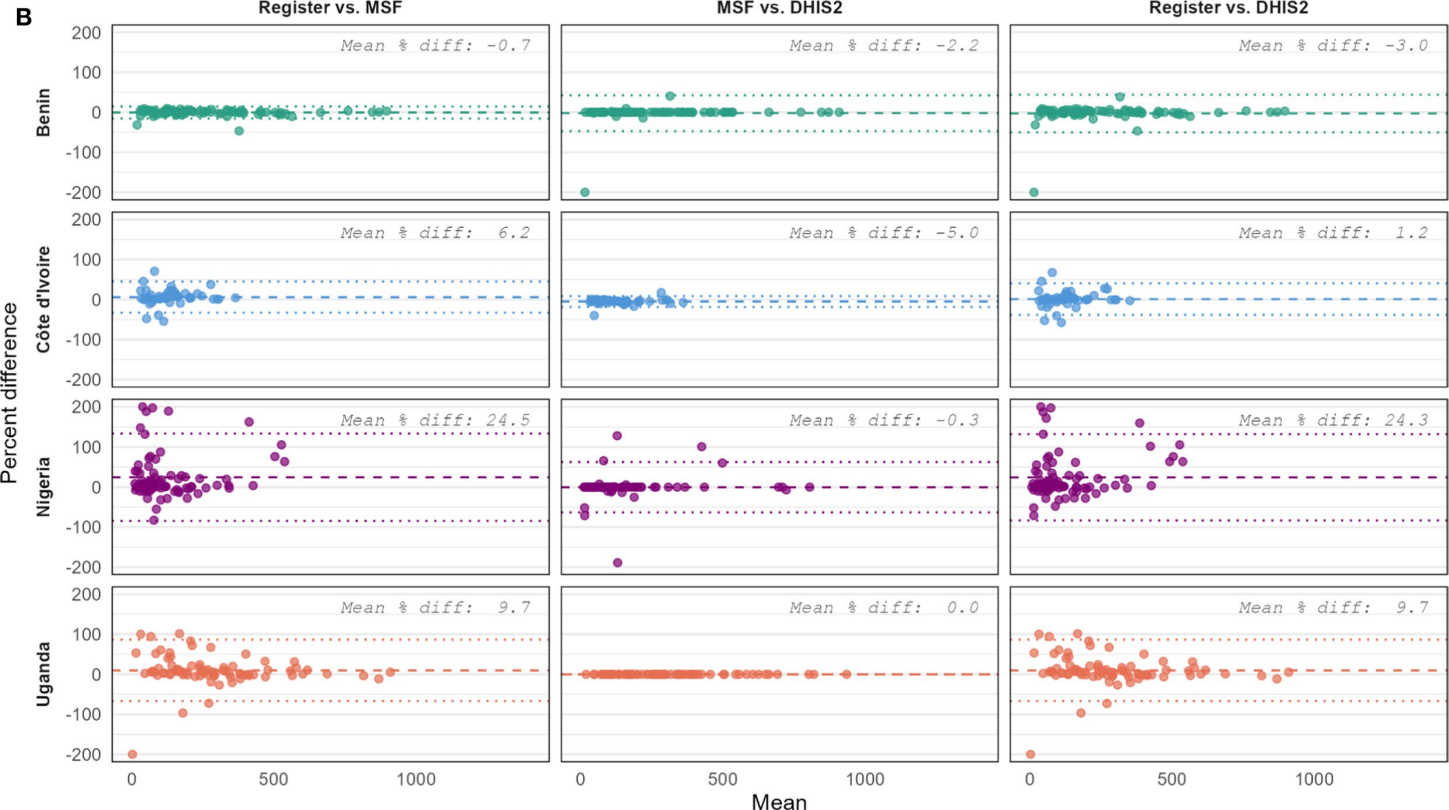
Bland–Altman plots comparing data sources for (**a**) total RDTs performed and (**b**) positive RDT results by country, 2023. Plots show the percentage difference between data sources against the mean number of RDTs performed per facility-month. Each panel represents a pairwise comparison of reporting sources (register vs. MSF, MSF vs. DHIS2, and register vs. DHIS2) stratified by country. The solid line indicates the mean percentage difference (bias), and the dotted lines represent the limits of agreement (mean ± 1.96 × standard deviation)

Data sources were largely concordant in Benin and Côte d’Ivoire, with relatively narrow LoA observed (Fig. 1). In contrast, greater variability and wider LoA were evident in data from Nigeria and Uganda. Across all countries, discrepancies were generally larger when comparing the health facility register to either aggregate data source than when comparing MSF to DHIS2. Most comparisons indicated that the aggregate data source overreported the indicator relative to the source document, with mean percentage differences as high as 24%. Overreporting was more pronounced for the number of positive RDT results than for the total number of RDTs performed. Visual inspection of the plots suggested that percentage differences tended to be greater when the number of RDTs performed or the number of positive results was low.

### WAPE-based aggregate data reporting accuracy

**Fig. 2:**
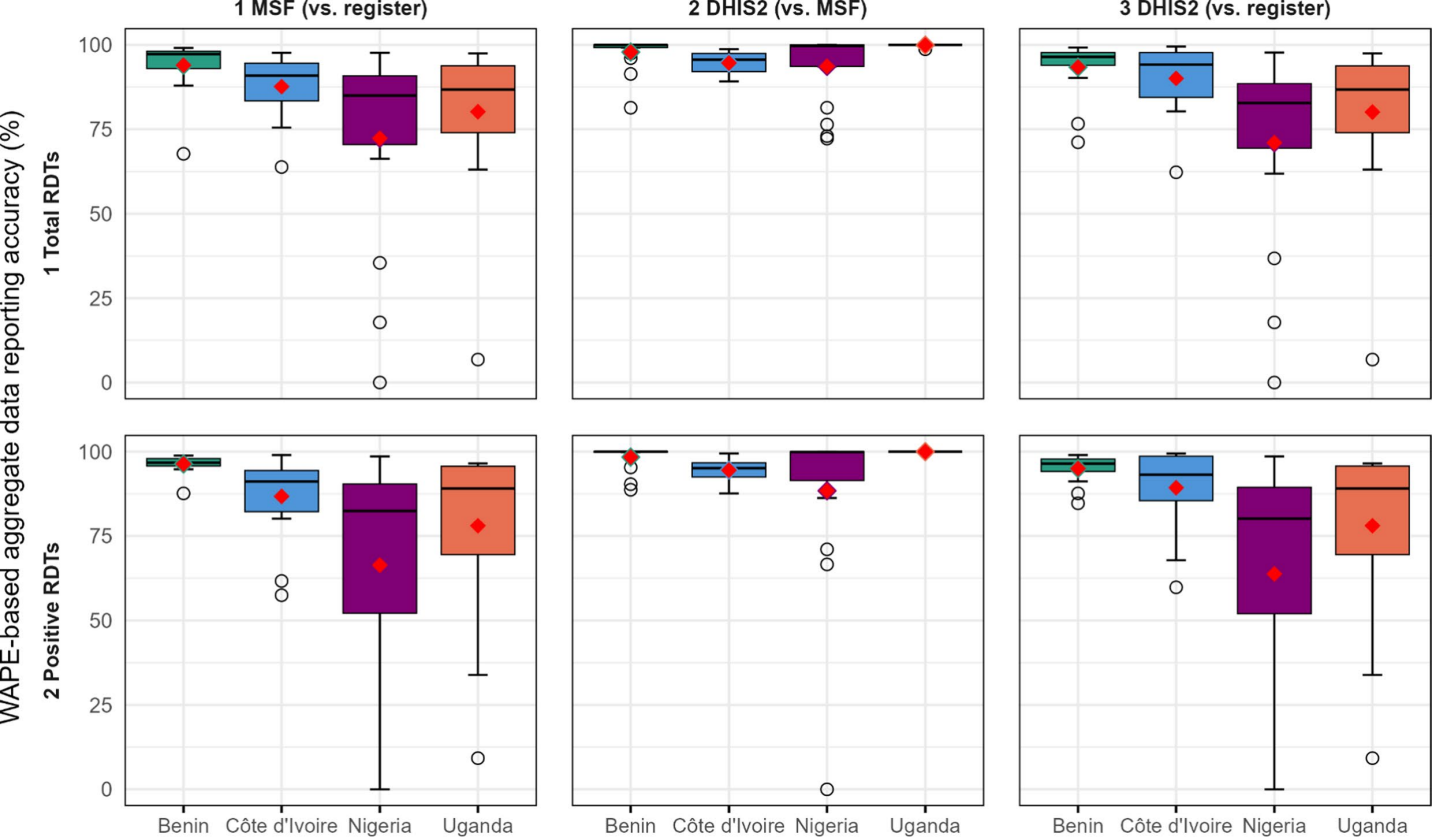
Distribution of the WAPE-based aggregate data reporting accuracy by comparison, indicator and country, 2023. Black lines indicate the median and red diamonds indicate the mean WAPE-based aggregate data reporting accuracy of 16 health facilities in each country. The box boundaries indicate the interquartile range, the whiskers identify the limits of 1.5 times the interquartile range and the hollow circles identify outliers

Data accuracy for both indicators, as measured by the WADRA, was highest when comparing MSF to DHIS2 across all four countries (Fig. 2). Both mean and median WADRA were similar for both indicators although the accuracy of total RDTs was higher for Nigeria than for positive RDTs. Among the countries, Benin again demonstrated the highest accuracy, with mean MSF accuracy compared to registers of 93.9% for total RDTs and 96.4% for positive RDTs. In contrast, Nigeria reported the lowest accuracy, with 72.3% and 66.4% accuracy for total and positive RDTs, respectively.

**Fig. 3:**
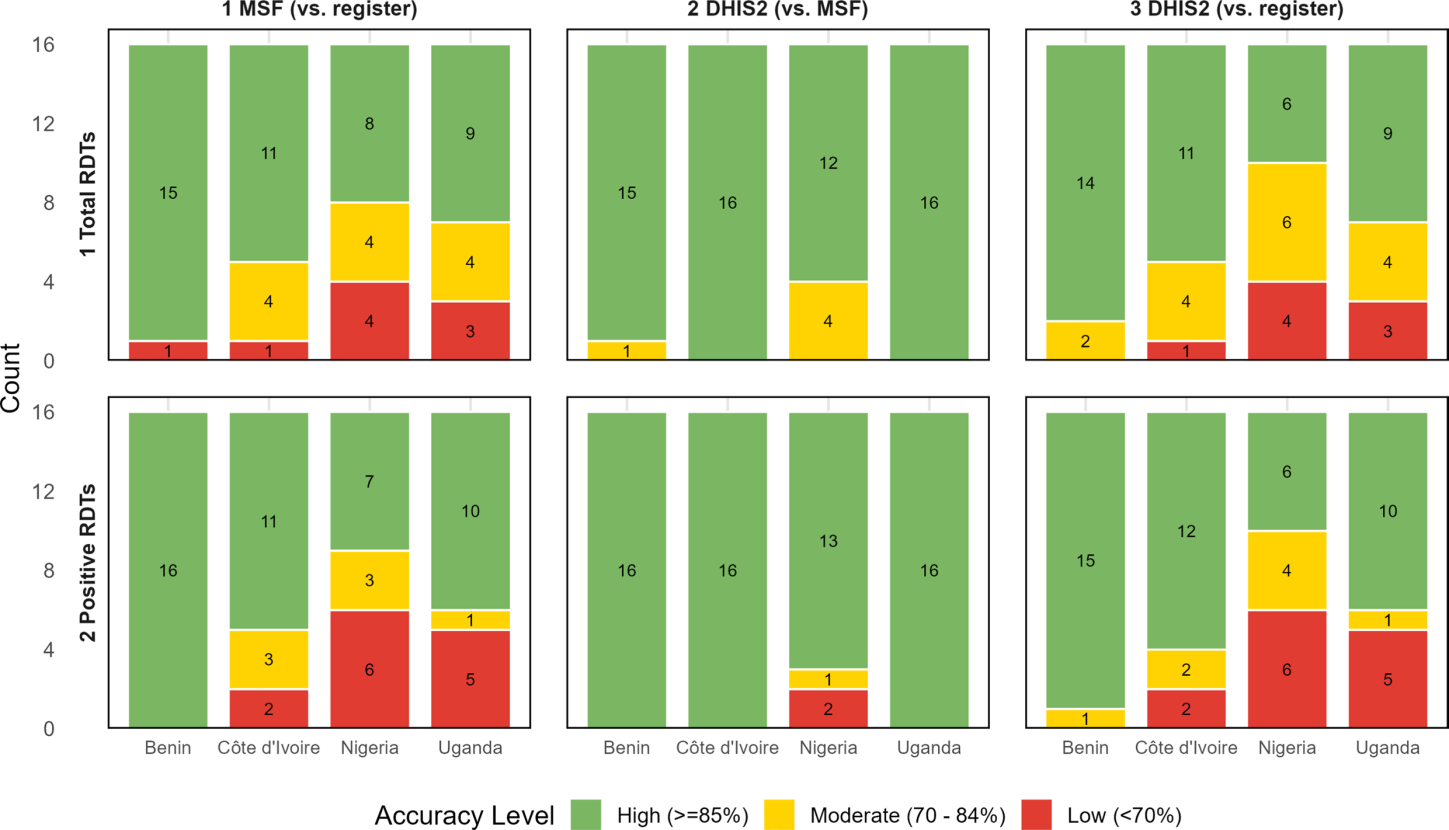
Number of health facilities with high, moderate or low levels of WAPE-based aggregate data reporting accuracy by comparison, indicator and country, 2023

The classification of the 16 health facilities per country according to their accuracy level demonstrated that in Benin between 14 and 16 (88%–100%) health facilities had high reporting accuracy (WADRA ≥ 85%) for both indicators across the three comparisons (Fig. 3). In Nigeria and Uganda, however, between 3 and 6 (19%–38%) facilities had low accuracy (WADRA < 70%), and only 6 to 10 (38%–62%) achieved high levels of accuracy in their aggregated reports.

### Verification factors

**Fig. 4:**
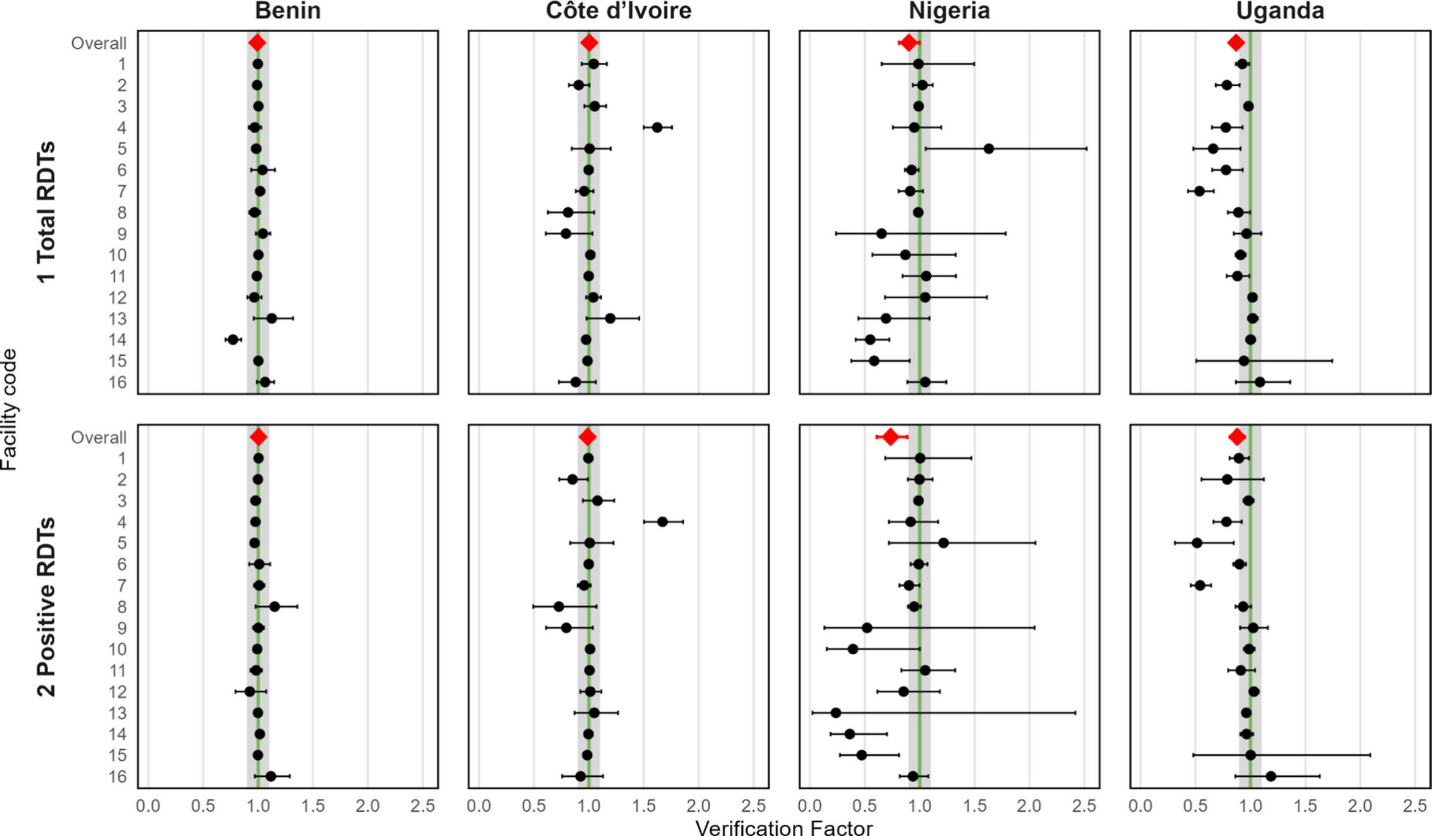
Forest plots of verification factors comparing total and positive RDTs between health facility registers and the DHIS2 by health facility and country, 2023. VF > 1 indicates that the HMIS underreports the indicator whereas VF < 1 indicates that the DHIS2 overreports the indicator compared to the register. Error bars indicate the 95% confidence interval. The shaded region indicates facilities with no serious reporting concerns, i.e. 0.9 ≤ VF < 1.1

VFs comparing the DHIS2 to the health facility registers further illustrated these trends (Fig. 4). In Benin and Côte d’Ivoire, average VFs suggested no important over- or underreporting at the aggregate level for either indicator, although individual health facilities could be identified with over- or underreporting. Greater inter-facility variability was observed in Côte d’Ivoire, including one facility with significant underreporting of both indicators and several facilities overreporting. In Nigeria, the VF for the number of total RDTs was within the acceptable boundary (0.90, 95% CI 0.81, 1.0) whereas the VF for positive RDTs indicated overreporting (0.74, 95% CI 0.61, 0.89). In Uganda, VFs for both total RDTs (VF 0.87, 95% CI 0.82, 0.92) and positive RDTs (VF 0.88, 0.82, 0.95) indicated overreporting. Both countries had a number of health facilities where DHIS2 data substantially overestimated register data for both indicators. However, the low number of RDTs performed at several facilities in Nigeria resulted in wide confidence intervals for the VFs.

### Association between region, baseline TPR and RDT volume and data concordance

Regional differences in WADRA were more marked in Nigeria than in Uganda, and greater for positive RDTs than total RDTs (Supplemental Fig. 1). The median WADRA for positive RDTs in health facilities in Oyo State in Nigeria was 86.0% compared to 42.7% in Sokoto State. The median WADRA for positive RDTs in the Busoga region was 79.1% compared to 92.8% in Lango. Regional differences were also clear from VFs (supplemental Fig. 2). Sokoto State in Nigeria overreported both total RDTs and positive RDTs whereas Oyo State showed no important reporting problem for either indicator. In Uganda, Busoga region overreported both total and positive RDTs whereas Lango had no reporting problem.

In Nigeria, mean WADRA was lower among strata with high outpatient volume compared to those with low volume (Supplemental Fig. 3). Differences by baseline TPR were less pronounced. A similar pattern was observed in Uganda, though differences between strata were smaller. Verification factors showed a similar trend in Nigeria but not in Uganda (Supplemental Fig. 4). In Nigeria, high-volume strata were associated with overreporting of both total and positive RDTs, although the 95% CI for the high TPR/high volume stratum for total RDTs included 1. Among the low-volume strata, total RDT reporting was either accurate or underreported, with both confidence intervals including 1. For positive RDTs, the high TPR/low volume stratum showed no major reporting issue, while the low TPR/low volume stratum was associated with overreporting. In contrast, VF patterns in Uganda did not appear to be associated with either baseline TPR or outpatient volume.

## Discussion

The quality of malaria surveillance data remains a bottleneck for good intervention planning at both the national and subnational levels. This multicountry evaluation of the concordance of RDT numbers and positive results between health facility registers, MSF and the DHIS2 showed important differences between countries, indicators and data sources. Overall, we observed the concordance of aggregated reports (both MSF and DHIS2) of RDTs compared to health facility registers was highest in Benin and lowest in Nigeria and Uganda, suggesting that health system differences may affect the quality of data on RDTs. Comparisons between the MSF and the DHIS2 generally showed greater concordance than either aggregated report with health facility registers, indicating that the data entry process is less problematic than the data aggregation process. Finally, the number of positive RDTs was more likely than total RDTs to be overreported by the MSF and the DHIS2, particularly in Nigeria.

The highest degree of concordance between all data sources was observed in Benin. This finding could be explained by recent efforts by the Ministry of Health of Benin to improve data quality throughout the country by implementing a novel data validation approach at health district level [12]. This validation process involves storing all RDT cassettes from health facilities and directly comparing them to health facility registers during monthly data validation meetings to ensure results have been accurately registered. A similar effort has been undertaken in some areas of Nigeria [13], and a recent evaluation has demonstrated the stability of RDT results on cassettes stored for one month [14].

The finding that the data on positive RDTs obtained from the DHIS2 were more likely to be overreported than total RDTs when compared to the health facility register has been reported previously from Zambia where they found incidence calculated from aggregated reports to be higher than that from health facility registers [7]. While 2 (12.5%) or fewer of the 16 facilities in Benin and Côte d’Ivoire were characterized as having a low WADRA accuracy level for positive RDTs, 6 (38%) facilities in Nigeria and 5 (31%) in Uganda were in that range. Both countries had an overall VF for positive RDTs < 0.9, indicating important overreporting.

In Nigeria and Uganda, regional differences were important. In Nigeria, facilities selected from Oyo State had higher levels of WADRA along with VFs that indicated no reporting problems, compared to Sokoto State that had a low level of WADRA along with VFs that indicated important overreporting, particularly of positive RDTs. Oyo State in Nigeria, but not Sokoto State, had implemented a monthly RDT validation process similar to the program in Benin [13]. It is possible that this monthly data validation could have played a role in improving concordance between health facility and DHIS2 RDT data in Oyo State. The regional differences in Uganda are less marked than in Nigeria but suggest that health system differences could be responsible for variation in reporting accuracy.

We also explored the potential effect of outpatient volume and TPR on data quality. In Nigeria, facilities with outpatient volumes that were higher than the median value of their surrounding facilities had lower levels of WADRA when comparing DHIS2 to facility data. VFs showed that lower accuracy in high patient volume strata was due to important overreporting of both total and positive RDTs. However, this finding was only partially replicated in Uganda, and facility variability in Benin and Côte d’Ivoire was too limited to explore these factors. There was no clear pattern related to baseline TPR, suggesting that the malaria burden may be less important than the health facility burden.

The explanation for why the data aggregation process, particularly in Nigeria, appears to increase the number of reported positive RDTs more than total RDTs remain unclear. Data aggregation is a manual procedure that involves tallying multiple entries, one at a time, for a large number of different variables. By default, the process is error prone, especially for high volume facilities. Overburdened health facilities may rely on ad hoc or temporary staff to manage the increased reporting demands, which could affect the accuracy of data transferred from registers to the MSF through manual aggregation [15]. It is also possible that data validation processes prior to entering data into DHIS2 may occasionally uncover RDT results that were not initially captured in the official registers, thereby increasing the number of positives. For instance, in some clinics, test outcomes noted in patient files may not always be transcribed into the register.

However, these explanations do not fully account for the tendency toward overreporting of positive RDTs. In a different evaluation of the accuracy of the recording of RDT results in registers in these same health facilities, we found that 5.0% to 7.1% of RDT results recorded in facility registers were negative tests misrecorded as positive [9]. One possible explanation for this finding is that health workers may intentionally misrecord negative results as positive, especially if doing so justifies the administration of antimalarial treatment. If treatment decisions do not align with the recorded test results, facility staff may feel pressure to reconcile discrepancies by adjusting the aggregated data. It is therefore possible that aggregated data may also, in some instances, be modified to align more closely with treatments provided rather than test outcomes alone.

Although VFs have been recommended by WHO for use in data quality audits, a recent report has highlighted important limitations to this method [16]. First, errors of over- and underreporting could cancel each other out in the VF, leading to a misclassification of the error rate. Secondly, the VF does not take into account the magnitude of the indicator and gives all health facilities equal weight, despite some facilities reporting significantly greater numbers of RDTs than others and contributing more to the degree of inaccuracy in the system. The WAPE method of measuring aggregate data reporting accuracy resolves many of these limitations as it uses absolute differences and weights results by the magnitude of the indicator. However, the WAPE and its associated accuracy measure are not able to indicate the direction of errors. Therefore, the VF could still play an important role in highlighting any patterns to under- or overreporting of indicators.

The study has several important limitations. The evaluation was conducted in only 16 public primary health care facilities per country, selected from specific geographic areas and limited to those with high DHIS2 reporting completeness. As a result, the findings may not be fully generalizable to all health facilities or regions within each country. Additionally, the limited number of facilities per country restricted the extent to which factors potentially influencing the magnitude or direction of discrepancies could be examined. Despite these limitations, the use of harmonized methods across four sub-Saharan African countries allowed for meaningful cross-country comparisons. The observed differences in data concordance may reflect underlying variations in health system processes, which could help identify context-specific opportunities to strengthen data quality.

## Conclusions

Notable discrepancies in RDT data between health facility registers and DHIS2 were observed in Nigeria and Uganda, and to a lesser extent in Côte d’Ivoire. In contrast, Benin demonstrated a high level of concordance across data sources. In Nigeria, discrepancies were more pronounced for positive RDT results than for total RDTs, and there was an exploratory association between higher outpatient volume and lower concordance between data sources. Further research, particularly in Nigeria and Uganda, is needed to better understand the system- and facility-level factors associated with low reporting accuracy. In addition, data validation approaches that compare recorded RDT results with physical RDT cassettes could be evaluated to assess their potential to improve the accuracy and reliability of routine malaria data.

## Supplementary Information

Additional file 1

## Data Availability

The datasets used and/or analyzed during the current study can be provided by the corresponding author on reasonable request.

## Abbreviations

DHIS2: District health information software 2
HMIS: Health management information system
MSF: Monthly summary form
OPD: Outpatient department
RDT: Rapid diagnostic test
TPT: Test positivity rate
