# Supplementary material for: Concordance of rapid diagnostic test results between health facility registers and health management information systems: a multi-country evaluation
Source: Malar J. 2025 Dec 31;25:70. doi: 10.1186/s12936-025-05753-4 (PMC12866365; doi:10.1186/s12936-025-05753-4)

Supplemental Table 1. Number of RDT tests performed, the number reported as positive and the test positivity rate by country, month and data source, 2023

| **Country** | **Month** | **Health facility register** | | | **MSF** | | | **DHIS2** | | |
| --- | --- | --- | --- | --- | --- | --- | --- | --- | --- | --- |
|  |  | **RDT tests performed**  **(n)** | **RDT-positive (n)** | **Test positivity rate**  **(%)** | **RDT tests performed**  **(n)** | **RDT-positive (n)** | **Test positivity rate**  **(%)** | **RDT tests performed**  **(n)** | **RDT-positive (n)** | **Test positivity rate**  **(%)** |
| Benin | July | 9529 | 5707 | 59.9 | 9718 | 5661 | 58.3 | 9718 | 5661 | 58.3 |
|  | August | 8186 | 4525 | 55.3 | 8119 | 4318 | 53.2 | 8195 | 4287 | 52.3 |
|  | September | 6798 | 3118 | 45.9 | 7066 | 3111 | 44.0 | 7135 | 3253 | 45.6 |
|  | October | 8260 | 3902 | 47.2 | 8198 | 3906 | 47.6 | 8090 | 3874 | 47.9 |
|  | November | 7920 | 3880 | 49.0 | 8008 | 3908 | 48.8 | 8016 | 3897 | 48.6 |
|  | **Total** | **40,693** | **21,132** | **51.9** | **41,109** | **20,904** | **50.9** | **41,154** | **20,972** | **51.0** |
| Côte d’Ivoire | September | 3455 | 2400 | 69.5 | 3644 | 2543 | 69.8 | 3522 | 2491 | 70.7 |
|  | October | 3310 | 2160 | 65.3 | 3493 | 2216 | 63.4 | 3232 | 2080 | 64.4 |
|  | November | 2702 | 1780 | 65.9 | 3016 | 2031 | 67.3 | 2858 | 1920 | 67.2 |
|  | **Total** | **9467** | **6340** | **67.0** | **10,153** | **6790** | **66.9** | **9612** | **6491** | **67.5** |
| Nigeria | July | 2932 | 1183 | 40.3 | 3874 | 2436 | 62.9 | 3310 | 2085 | 63.0 |
|  | August | 3951 | 1950 | 49.4 | 4067 | 2324 | 57.1 | 3937 | 2398 | 60.9 |
|  | September | 4281 | 2342 | 54.7 | 4544 | 2708 | 59.6 | 4526 | 2909 | 64.3 |
|  | October | 3680 | 1935 | 52.6 | 4567 | 2690 | 58.9 | 4487 | 2952 | 65.8 |
|  | November | 3001 | 1331 | 44.4 | 3455 | 1577 | 45.6 | 3136 | 1480 | 47.2 |
|  | **Total** | **17,845** | **8741** | **49.0** | **20,507** | **11,735** | **57.2** | **19,396** | **11,824** | **61.0** |
| Uganda | July | 9635 | 6593 | 68.4 | 10,493 | 6603 | 62.9 | 10,493 | 6602 | 62.9 |
|  | August | 7243 | 4530 | 62.5 | 8772 | 5087 | 58.0 | 8773 | 5087 | 58.0 |
|  | September | 5577 | 3333 | 59.8 | 6128 | 3510 | 57.3 | 6068 | 3510 | 57.8 |
|  | October | 6117 | 3261 | 53.3 | 6536 | 3568 | 54.6 | 6538 | 3569 | 54.6 |
|  | November | 7819 | 4374 | 55.9 | 8737 | 4706 | 53.9 | 8737 | 4706 | 53.9 |
|  | **Total** | **36,391** | **22,091** | **60.7** | **40,666** | **23,474** | **57.7** | **40,609** | **23,474** | **57.8** |
|  | **Overall** | **104,396** | **58,304** | **55.8** | **112,435** | **62,903** | **55.9** | **110,771** | **62,761** | **56.7** |

DHIS2: district health information system 2; MSF: monthly summary form; RDT: rapid diagnostic test.

Supplemental figure 1. Distribution of facility WAPE-based aggregate data reporting accuracy by region and indicator, Nigeria and Uganda, 2023


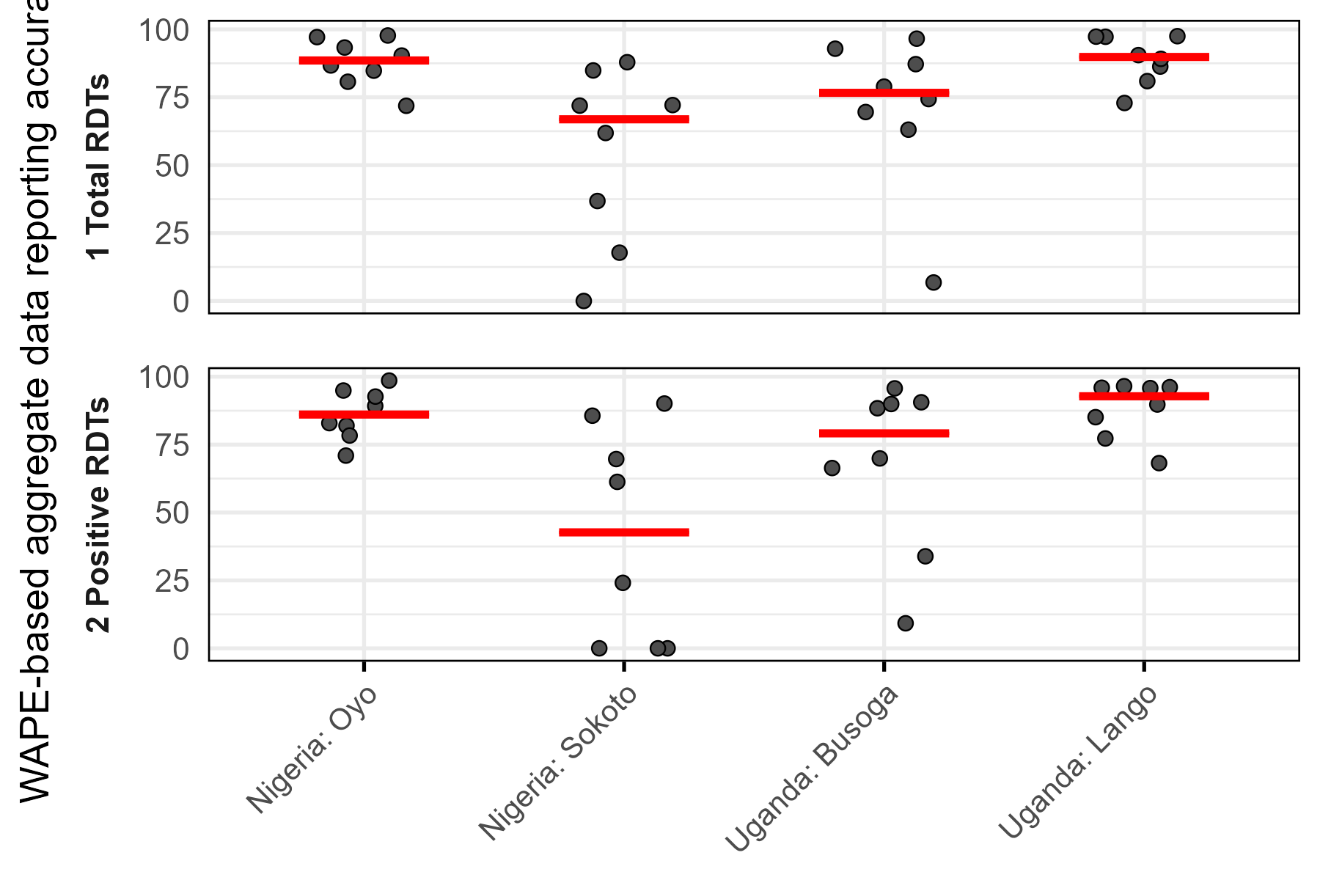


RDTs: rapid diagnostic tests. Red lines indicate the median value.

Supplemental figure 2. Verification factors by country, region and indicator, Nigeria and Uganda, 2023


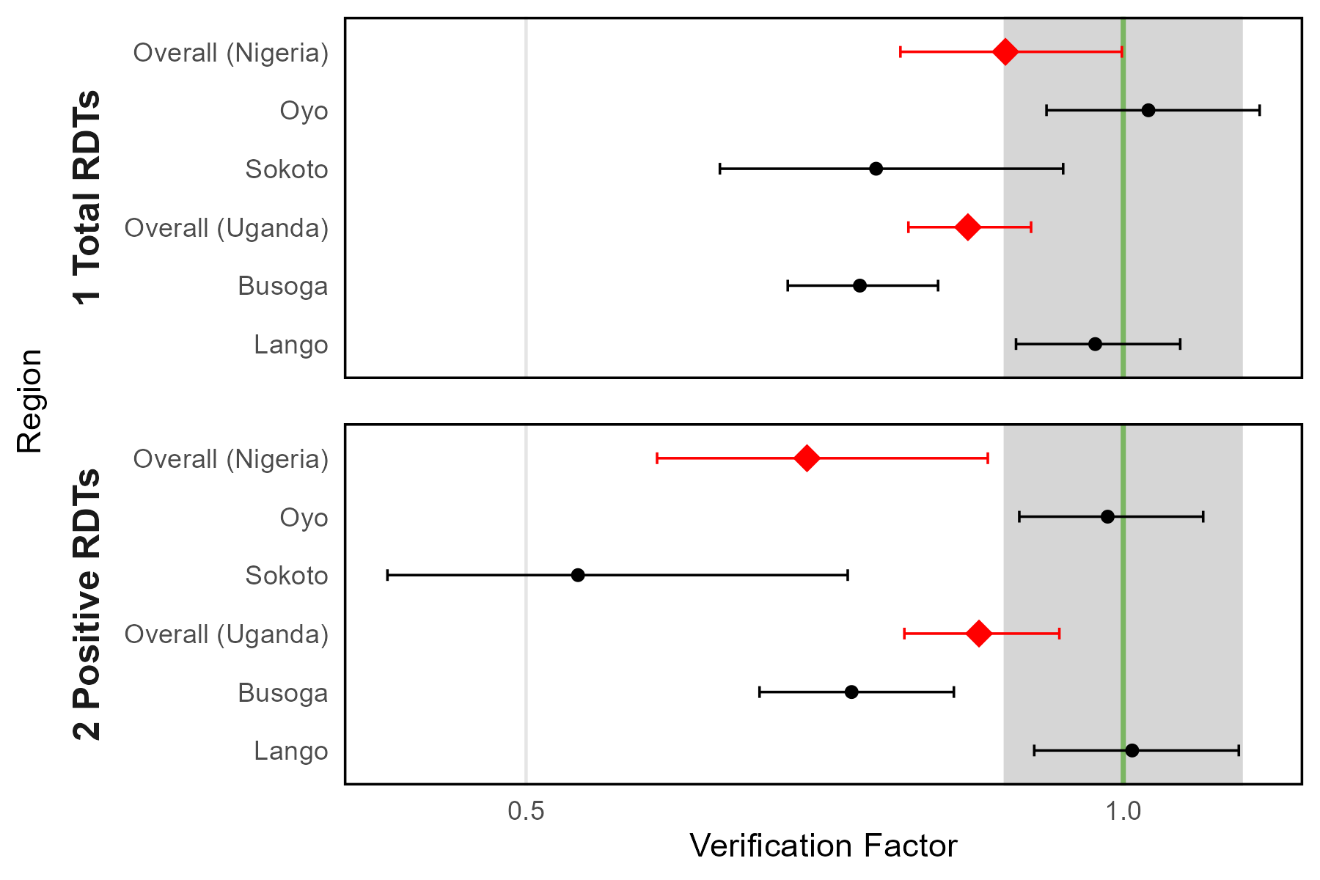


Supplemental Figure 3. Distribution of WAPE-based aggregate data reporting accuracy by baseline strata, country and indicator, Nigeria and Uganda, 2023


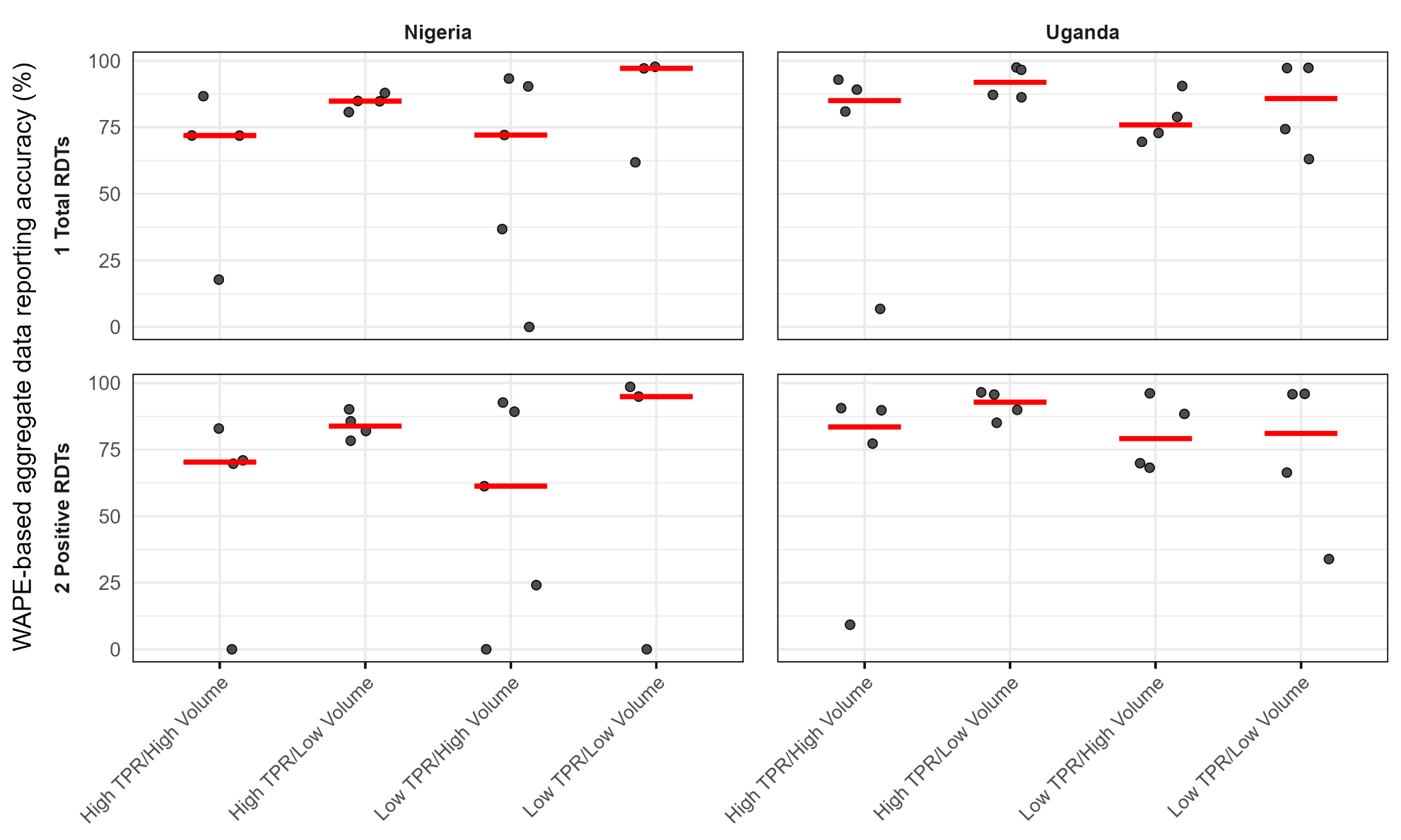


Supplemental Figure 4. Verification factors by baseline strata, country and indicator, Nigeria and Uganda, 2023


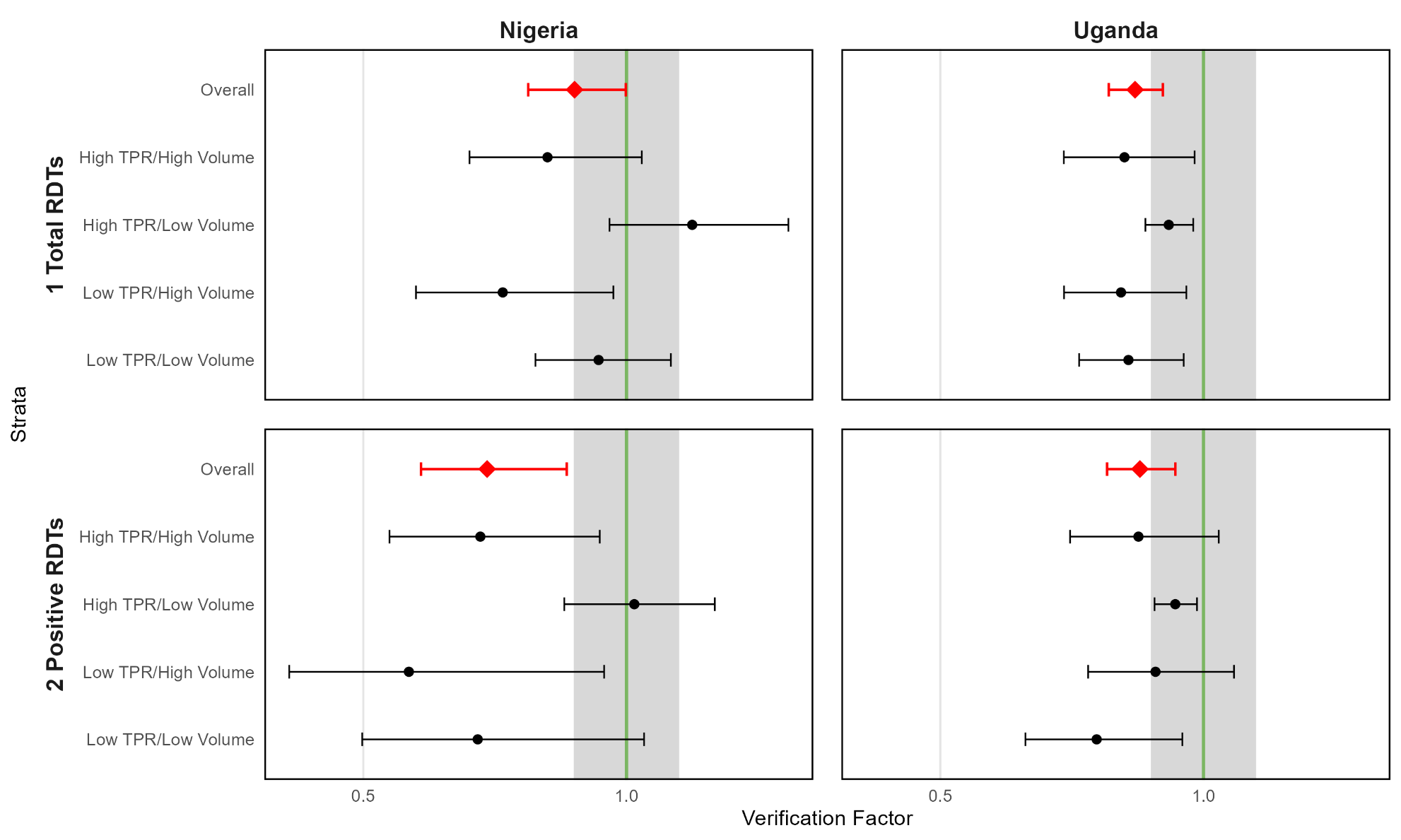

Supplement: Supplementary file 1 — Additional file 1 [file 12936_2025_5753_MOESM1_ESM.docx]
